# Investigating obesity as a risk factor for influenza‐like illness during the 2009 H1N1 influenza pandemic using the Health Survey for England

**DOI:** 10.1111/irv.12420

**Published:** 2016-08-20

**Authors:** Rachel Murphy, Ellen B. Fragaszy, Andrew C. Hayward, Charlotte Warren‐Gash

**Affiliations:** ^1^ London School of Hygiene & Tropical MedicineLondonUK; ^2^ Institute of Health InformaticsUniversity College LondonLondonUK; ^3^ Present address: The Farr Institute of Health Informatics ResearchLondonUK

**Keywords:** body mass index, Health Survey for England, influenza‐like illness, obesity

## Abstract

**Background:**

Following the 2009 H1N1 influenza pandemic, obesity was shown to be associated with severe influenza outcomes. It remains unclear whether obesity was a risk factor for milder influenza‐like illness (ILI).

**Objectives:**

To determine whether obesity was associated with an increased risk of self‐reported ILI during the 2009 H1N1 influenza pandemic using Health Survey for England (HSE) 2010 cross‐sectional data.

**Methods:**

This study used HSE data collected from English households between January and December 2010. Weight and height measurements were taken by trained fieldworkers to determine obesity. ILI was defined as a positive response to the question “Have you had a flu‐like illness where you felt feverish and had a cough or sore throat?” with illness occurring between May and December 2009. Multivariable logistic regression was used to evaluate the association between obesity and ILI.

**Results:**

The study comprised 8407 participants (6984 adults, 1436 children), among whom 24.7% (95% CI: 23.6–25.9) were classified as obese. Of obese participants, 12.8% (95% CI: 11.1–14.8) reported ILI compared to 11.8% (95% CI: 10.8–12.8) of non‐obese participants. The adjusted OR for ILI associated with obesity was 1.16 (95% CI: 0.98–1.38, *P*=.093). For adults and children, the adjusted ORs were 1.16 (95% CI: 0.97–1.38, *P*=.101) and 1.26 (95% CI: 0.72–2.21, *P*=.422), respectively.

**Conclusion:**

Household survey data showed no evidence that obesity was associated with an increase in self‐reported ILI during the 2009 H1N1 influenza pandemic in England. Further studies using active prospective ILI surveillance combined with laboratory reporting would reduce bias and improve accuracy of outcome measurements.

## 1 Introduction

New evidence emerged following the 2009 H1N1 influenza pandemic that obesity was an independent risk factor for hospitalization, intensive care unit admission and death in laboratory‐confirmed influenza cases from 19 countries.^1^ Obesity has also been shown to aggravate the effect of seasonal influenza on respiratory mortality independent of the effect of comorbidities and meteorological factors.^2^ Although obesity is associated with immune dysregulation at the cellular level,^3^, ^4^ it remains unclear whether it is associated with severe influenza outcomes through increasing the risk of acquiring influenza infection, promoting progression to severe disease after infection or both.^5^, ^6^

The vast majority of people with symptomatic influenza have mild illnesses and therefore do not seek medical attention^7^; rates of laboratory testing increase with severity of illness. Studies based on medical records and/or laboratory testing thus fail to capture much of the burden of community influenza‐like illness (ILI)^8^ and may not yield insights transferable to the wider population. Studies using self‐reported height and weight measurements to calculate body mass index (BMI) may be subject to recall bias and misclassification due to participants reporting a more socially desirable height and weight.^9^ Other studies based on BMI measurements extracted from medical records may be limited by lack of timeliness and accuracy of BMI recording.

In this study, we use data from the Health Survey for England (HSE)^10^—a nationally representative population‐based study in which obesity classification is based on weight and height measurements taken by trained fieldworkers—to determine whether obesity was associated with an increased risk of self‐reported ILI during the 2009 H1N1 influenza pandemic.

## 2 Methods

### 2.1 Data source and population

The 2010 HSE data set was used for this study.^11^ It comprised data from a sample of adults aged 16+ years and children 0 to 15 years representative of private households in England. HSE methods are described elsewhere.^12^ All data used in our study, including detailed information on social and demographic characteristics, lifestyle behaviours, health and physical measurements such as weight and height, were collected during household visits by trained interviewers, which took place throughout the 2010 calendar year. For inclusion in this study, HSE participants had to have both a valid measure of obesity and a valid response to the question used to identify influenza‐like illness.

### 2.2 Definition of outcome and exposure

The outcome of interest was ILI experienced during the first eight months of the H1N1 pandemic between May 2009 and December 2009. The question used to identify ILI was “Since May 2009, have you had a flu‐like illness where you felt feverish and had a cough or sore throat?” Permitted responses were “yes” and “no” with the responses “don't know” and “refused” recoded to missing. If participants responded “yes,” they were asked to report the month and year of illness. Illnesses reported to occur after December 2009 were not included in the outcome definition.

The primary exposure, obesity, was based on BMI measurements for adults aged 16 and over and on age‐ and sex‐based population centiles for children aged 2–15 years. Those aged under two did not have height measurements so were excluded from analyses. Interviewers measured height on those aged 2+ and weight for all participants. For participants exceeding the weight limit of the scales (130 kg), self‐reported weights were used to calculate BMI. BMI measurements considered unreliable (i.e. pregnant women, those who refused to be measured, measurements that were attempted but not obtained, measurements that were not attempted or measurements that were not useable) were excluded from analyses. Adult participants with a BMI 30 kg/m^2^ or above were classified as obese; children were classified as obese if their weight exceeded the 95th centile.^13^ A binary variable for obesity (obese vs non‐obese) was generated that combined results for adults and children. We also conducted two sensitivity analyses: first we reclassified BMI for adults as underweight/normal (BMI <25), overweight (BMI 25–29.999) and obese (BMI≥30), then removed the overweight category to compare obese with underweight/normal weight individuals; second, we used the waist hip ratio variable as an alternate measure of obesity in adults only, which was classified as either “not at increased risk” or “substantially increased risk” according to standard cut‐offs.^14^ Only participants with a valid waist hip ratio measurement were included in this analysis.

### 2.3 Potential confounding and effect modifying variables

We considered variables as potential confounders or effect modifiers based on findings from previous studies and consideration of plausible biological mechanisms. At individual level across the whole study population, we considered age, sex, ethnicity, receiving influenza vaccination from October 2009 (the month the pandemic vaccination was introduced in the UK), clinician‐diagnosed asthma and season of interview. Information on some potential confounding variables was available for adults only. These included smoking status (reported as current smoker, ex‐smoker or never smoker), frequency of alcohol consumption in the last 12 months, clinician diagnosis of chronic obstructive pulmonary disease (COPD), high blood pressure or diabetes and level of education achieved. The household level variables urbanization, household size and Index of Multiple Deprivation 2007 (IMD2007) were also included.

### 2.4 Statistical analysis

Data analysis was conducted using Stata version 13.0 (Stata Corporation, TX, USA). We described baseline characteristics of participants by calculating the frequency and survey weighted percentage for all categories of variables of interest. Weighting was used to correct the distribution of household members to match population estimates for sex/age groups and geographic region, as well as to correct for bias resulting from individual non‐response within households.^15^ Stata's “svy” suite of commands was used to account for the complex survey design. We used univariate logistic regression analysis to generate an odds ratio with 95% confidence interval for the association between obesity and influenza‐like illness, with participants who were not obese (BMI <30 kg/m^2^) forming the baseline reference group. Univariate associations between all potential confounders and obesity and separately ILI were investigated through chi‐squared tests and logistic regression analysis. We also drew a causal diagram using the programme DAGitty v 2.3 (http://www.dagitty.net/dags.html#) to inform choice of variables for inclusion in multivariable logistic regression models (see Supplementary material). Multivariable models were then generated: the main model included all theoretically relevant confounders associated with both outcome and exposure, for which adjustment was identified as necessary using our causal diagram. Each variable added was examined for multicollinearity. For asthma, COPD and smoking, we generated a composite binary variable which was positive if any of these variables was positive. For each model, we examined the change in effect size and the Wald test *P*‐value compared to the crude model. Potential effect modifiers identified based on existing literature (influenza vaccination, type of diabetes and age) were also evaluated using interaction terms within the final logistic regression model. The Wald test *P*‐value was used to assess the strength of the interaction. Analyses were repeated separately for adults and children. Other sensitivity analyses were conducted for adults only to investigate the effect of varying the definition of obesity. These were 1) removing the overweight category to compare obese individuals with normal/underweight individuals and 2) using waist–hip ratios in place of BMI as a measure of obesity.

## 3 Results

### 3.1 Participant characteristics

**Figure 1:**
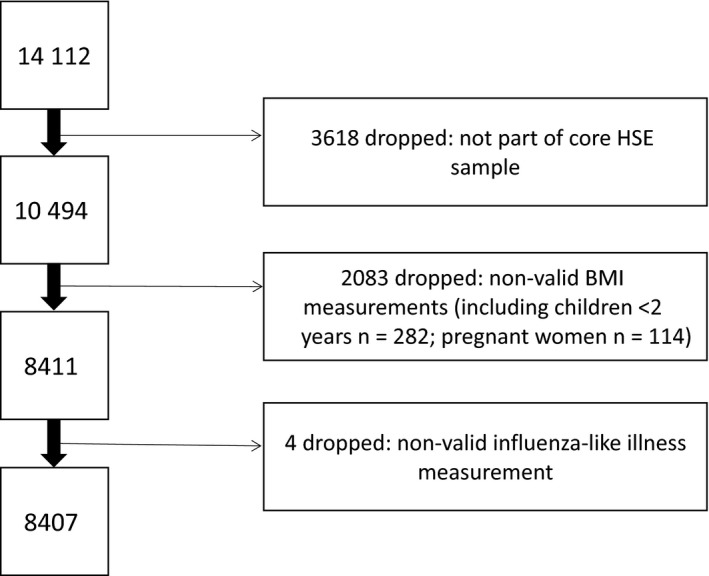
Flow diagram of study participants

Between January and December 2010, the HSE selected 8736 households, of which 90.8% met the inclusion criteria and 66% participated. From the core HSE sample of 10 494 participants interviewed, 2803 participants were excluded due to non‐valid BMI measurements and a further four because of invalid influenza‐like illness responses, leaving 8407 participants in our study (Fig. 1).

These were 6984 adults with a median age of 48 years (IQR 35–63 years), and 1436 children with a median age of 9 years (IQR 5–12 years). There were 3872 males and 4535 females in the data set. Overall, 2159 participants, a weighted percentage of 24.7% (95% CI: 23.6–25.9), were classified as obese. For adults, corresponding figures were 26.1% (95% CI: 24.9–27.4) compared to 17.3% (95% CI: 1 5.2–19.7) for children. A total of 197 adults (2.8%) were morbidly obese (BMI >40). In total, 996 participants (12.0% [95% CI: 11.1–13.0]) reported ILI between May and December 2009. Baseline characteristics of participants presented by obesity category are shown in Table 1.

**Table 1:** Baseline characteristics of study population, n=8407

Variable	Category	Total number	Number obese	Number not obese	Weighted percentage (95% CI)
Age group (years)	Under 5	315	41	274	3.3 (2.9–3.7)
	5–14.9	1108	210	898	13.0 (12.2–13.9)
	16–24.9	721	90	631	12.6 (11.3–13.9)
	25–34.9	989	208	781	13.6 (12.6–14.7)
	35–44.9	1246	336	910	15.3 (14.4–16.3)
	45–54.9	1283	400	883	14.8 (14.0–15.6)
	55–64.9	1165	394	771	12.4 (11.5–13.2)
	65 and over	1580	480	1100	15.1 (14.1–16.1)
Sex	Male	3872	1011	2861	50.3 (49.2–51.3)
	Female	4535	1148	3387	49.8 (48.7–50.8)
Ethnicity	White	7515	1951	5564	87.06 (85.2–88.7)
	Mixed	147	29	118	1.8 (1.4–2.3)
	Asian	445	89	356	6.7 (5.5–8.1)
	Black	216	67	149	3.2 (2.5–4.1)
	Other	74	21	53	1.2 (0.8–1.8)
Obesity	Yes	2159	2159	0	24.7 (23.6–25.88)
	No	6248	0	6248	75.3 (74.1–76.4)
ILI between May and December 2009	Yes	996	270	726	12.0 (11.1–13.0)
	No	7411	1889	5522	88.0 (87–88.9)
Self‐reported influenza vaccination from October 2009	Yes	2254	737	1517	23.4 (22.3–24.6)
	No	6147	1421	4726	76.6 (75.4–77.7)
High blood pressure^a^ (adults only)	Yes	1906	823	1083	23.8 (22.6–24.9)
	No	5061	1083	3978	76.2 (75.1–77.4)
Diabetes^a^ (adults only)	Yes	447	239	208	5.7 (5.1–6.3)
	No	6532	1667	4865	94.3 (93.7–94.9)
Asthma^a^	Yes	1393	390	1003	16.7 (15.8–17.7)
	No	7012	1769	5243	83.3 (82.4–84.2)
Interview season	Spring (March–May)	2293	579	1714	27.2 (23.7–31.0)
	Summer (June–August)	2078	550	1528	24.7 (21.3–28.4)
	Autumn (September–November)	2200	553	1647	26.1 (22.7–29.8)
	Winter (December–February)	1836	477	1359	22.1 (19.0–25.5)
Smoking status (adults only)	Current smoker	1371	336	1035	20.5 (19.3–21.9)
	Ex‐smoker	1886	620	1266	24.8 (23.6–26.0)
	Never smoker	3705	951	2754	54.7 (53.1–56.3)
Alcohol consumption in the last 12 mo (adults only)	At least weekly	4041	1000	3041	57.3 (55.6–59.0)
	At least monthly	950	278	672	14.2 (13.3–15.2)
	At least yearly	1115	383	732	15.6 (14.5–16.7)
	Not at all in last yr	855	246	609	12.9 (11.7–14.2)
COPD^a^ (adults only)	Yes	363	127	236	4.6 (4.1–5.1)
	No	6614	1780	4834	95.4 (94.9–96.0)
Level of education achieved (adults only)	Degree	1548	322	1226	23.2 (21.9–24.5)
	School	3800	1034	2766	56.1 (54.7–57.5)
	Foreign/Other	117	30	87	1.4 (1.2–1.7)
	No qualification	1510	520	1179	19.4 (18.2–20.7)
Urbanization^b^	Urban	3586	1056	2530	81.1 (78.4–83.4)
	Town and fringes	447	139	308	9.0 (7.5–10.9)
	Village, hamlet and isolated dwelling	482	121	361	9.9 (8.4–11.7)
Household size (persons)^b^	One	1197	364	833	27.6 (26.0–29.2)
	Two	1656	494	1162	35.7 (34.3–37.1)
	Three to five	1580	433	1147	35.0 (33.6–36.6)
	Six or more	82	25	57	1.8 (1.4–2.2)
IMD 2007^b^ (least to most deprived)	0.37–8.32	1013	240	773	21.6 (19.4–24.0)
	8.32 to >13.74	897	234	663	19.4 (17.6–21.4)
	13.74 to >21.22	894	268	626	20.3 (18.4–22.4)
	21.22 to >34.42	888	294	594	19.8 (18.0–21.8)
	34.42 to>85.46	823	280	543	18.8 (16.8–21.0)

a Self‐report of clinician diagnosis.

b Household level variable, n=4515.

### 3.2 Univariate analysis

Among obese participants, 12.8% (95% CI: 11.1–14.8) experienced ILI between May to December 2009 compared to 11.8% (95% CI: 10. 8–12.8) of participants who were not obese. The unadjusted OR was 1.11 (95% CI: 0.93–1.31, *P*‐value: .241). The highest odds of reporting ILI were seen in the age group 25–34.9 years, and ILI was least reported among people aged 65 years and over. Asthma, COPD and current smoking were associated with an increase in ILI reporting on univariate analysis, while hypertension was associated with a small decrease. The frequency of ILI reporting also varied by interview season and was most common in spring. People with no qualifications or foreign/other qualifications reported less ILI than those with higher levels of education. Obesity was associated with age, ethnicity, influenza vaccination, high blood pressure, diabetes, asthma, smoking status in adults, alcohol consumption in adults, COPD, household size, IMD score and education on univariate analysis (Table 2).

**Table 2:** Odds ratios for associations between potential confounding factors, ILI and obesity

Variable	Category	OR for association with ILI (95% CI)	Wald *P*‐value	OR for association with obesity (95% CI)	Wald *P*‐value
Age group	Under 5	1	<.0001	1	<.0001
	5–14.9	0.68 (0.44–1.03)		1.44 (0.97–2.14)	
	16–24.9	1.11 (0.71–1.74)		0.88 (0.56–1.37)	
	25–34.9	1.30 (0.86–1.97)		1.64 (1.12–2.39)	
	35–44.9	1.18 (0.80–1.74)		2.37 (1.61–3.48)	
	45–54.9	0.95 (0.62–1.44)		3.08 (2.10–4.51)	
	55–64.9	0.92 (0.60–1.40)		3.34 (2.26–4.94)	
	65 and over	0.41 (0.27–0.64)		2.76 (1.87–4.06)	
Sex	Male	1	.326	1	.531
	Female	1.07 (0.93–1.24)		0.97 (0.88–1.07)	
Ethnicity	White	0.52 (0.17–1.62)	.520	0.73 (0.36–1.46)	.025
	Mixed	0.62 (0.18–2.10)		0.55 (0.24–1.25)	
	Asian	0.61 (0.19–1.96)		0.53 (0.25–1.13)	
	Black	0.40 (0.11–1.44)		0.94 (0.41–2.15)	
	Other	1		1	
Self‐reported influenza vaccination from Oct 2009	Yes	1.02 (0.86–1.22)	.804	1.67 (1.49–1.88)	<.0001
	No	1		1	
High blood pressure^a^	Yes	0.80 (0.68–0.96)	.014	3.09 (2.74–3.49)	<.0001
	No	1		1	
Diabetes^a^	Yes	0.95 (0.71–1.26)	.761	3.48 (2.85–4.25)	<.0001
	No	1		1	
Asthma^a^	Yes	1.47 (1.24–	<.0001	1.15 (1.01–1.31)	.042
	No	1.74) 1		1	
Interview season	Spring (March–May)	1	<.0001	1	.838
	Summer (June–August)	0.69 (0.55–0.86)		1.06 (0.90–1.24)	
	Autumn (September–November)	0.58 (0.45–0.75)		1.02 (0.86–1.21)	
	Winter (December–February)	0.95 90.75–1.21)		1.07 (0.90–1.27)	
Smoking status (adults only)	Current smoker	1.24 (1.02–1.50)	.031	0.95 (0.81–1.13)	<.0001
	Ex‐smoker	0.93 (0.77–1.12)		1.52 (1.33–1.74)	
	Never smoker	1		1	
Alcohol consumption in the last 12 mo (adults only)	At least weekly	0.95 (0.74–1.22)	.348	0.84 (0.70–1.02)	<.0001
	At least monthly	1.11 (0.82–1.50)		1.01 (0.80–1.27)	
	At least yearly	1.11 (0.83–1.49)		1.29 (1.05–1.58)	
	Not at all in the last year	1		1	
COPD^a^ (adults only)	Yes	1.41 (1.04–1.91)	.026	1.54 (1.20–1.97)	.0007
	No	1		1	
Level of education achieved (adults only)	Degree	1	<.0001	1	<.0001
	School	1.00 (0.82–1.22)		1.41 (1.20–1.66)	
	Foreign/Other	0.32 (0.14–0.75)		1.34 (0.86–2.09)	
	No qualification	0.60 (0.46–0.78)		1.97 (1.65–2.35)	
Urbanization^b^	Urban	1	.195	1	.249
	Town and fringes	0.96 (0.70–1.32)		1.13 (0.93–1.38)	
	Village, hamlet and isolated dwelling	0.76 (0.57–1.02)		0.92 (0.77–1.10)	
Household size (persons)^b^	One	1	.036	1	<.0001
	Two	0.74 (0.60–0.92)		0.93 (0.79–1.09)	
	Three to five	0.83 (0.67–1.04)		0.69 (0.59–0.80)	
	Six or more	1.08 (0.66–1.77)		0.61 (0.41–0.91)	
IMD 2007^b^	0.37–8.32	1	.441	1	<.0001
	8.32 to >13.74	0.92 (0.72–1.19)		1.08 (0.88–1.32)	
	13.74 to >21.22	1.08 (0.84–1.39)		1.26 (1.05–1.52)	
	21.22 to >34.42	1.18 (0.92–1.51)		1.43 (1.19–1.72)	
	34.42 to >85.46 (most deprived)	1.08 (0.81–1.43)		1.56 (1.27–1.90)	

a Self‐report of clinician diagnosis.

b Household level variable, n=4515.

### 3.3 Multivariable analysis

In multivariable analysis across the whole study population (adults and children), the adjusted OR for the effect of obesity on likelihood of ILI was 1.16 (95% CI 0.98–1.38) *P*=.093. In an adults ‐only model with additional adjustment for highest educational qualification, the adjusted OR was 1.16 (95% CI 0.97–1.38) (*P*=.101). Similar effects were seen in a child‐only model—adjusted OR 1.26 (95% CI 0.72–2.21) (*P*=.422). There was no evidence of interaction in these models.

### 3.4 Sensitivity analyses

In sensitivity analyses of adults only, removing the overweight category (n=2627) to compare ILI reporting in obese adults (n=1908) with normal/underweight adults (n=2449) resulted in an adjusted OR of 1.14 (95% CI 0.92–1.40) *P*=.222. A second sensitivity analysis to investigate ILI reporting in adults with high waist–hip ratios (n=1979) compared with adults with normal waist–hip ratios (n=2932) again showed that there was little difference—adjusted OR 1.08 (95% CI 0.88–1.32) (*P*=.472). Results of multivariable models and sensitivity analyses are shown in Table 3.

**Table 3:** Effect estimates of total population and subpopulation analysis on the association between obesity and ILI

Participants	Obesity category	Crude OR (95% CI)	Wald test *P*‐value	Adjusted OR^a^ (95% CI)	Wald test *P*‐value
All	Non‐obese	1	–	1	–
	Obese	1.11 (0.93–1.31)	.241	1.16 (0.98–1.38)	.093
Adults only	Non‐obese	1	–	1	–
	Obese	1.07 (0.91–1.27)	.417	1.16 (0.97–1.38)	.101
Children only	Non‐obese	1	–	1	–
	Obese	1.23 (0.71–2.15)	.461	1.26 (0.72–2.21)	.422
Sensitivity analysis 1 (adults only, excluding overweight category)	Non‐obese	1	–	1	–
	Obese	1.03 (0.85–1.25)	.735	1.14 (0.92–1.40)	.222
Sensitivity analysis 1 (adults only, using waist–hip ratio)	Non‐obese	1	–	1	–
	Obese	0.88 (0.73–1.06)	.167	1.08 (0.88–1.32)	.472

a Adjusted for age, sex, household size, composite lung variable (asthma, COPD, smoking) and, for adult model only, education.

## 4 Discussion

We found no evidence that obesity was associated with an increase in self‐reported ILI during the 2009 H1N1 pandemic in English households using representative population data from the HSE. In our data set, people aged 25–35 were most likely to experience ILI and the over 65 age group were least likely to report ILI, consistent with other data from the pandemic.^8^ We have previously shown that ILIs reported in the HSE 2010 show a similar pattern and age distribution to infections identified in the Flu Watch cohort study,^16^ although the overall magnitude of ILI was considerably less. The Flu Watch cohort was designed to estimate the community burden of ILI by collecting data using active weekly prospective follow‐up but did not measure obesity.

Meta‐analysis of hospitalization and death data from the 2009 pandemic suggests that obesity is an independent risk factor for severe outcomes of pandemic influenza.^1^ Individual studies report similar findings for seasonal influenza.^2^, ^17^, ^18^ One case cohort study found that obese adults aged 20–59 years had an increased risk of attending outpatient clinics with ILI symptoms than those of normal weight in the influenza seasons 2004/5 and the 2009 pandemic.^19^ An Australian population health survey showed that people with obesity were more likely to report ILI during July to September 2009 than people of normal weight.^20^ Few other studies, though, have investigated the role of obesity as a risk factor for mild ILI in the community.

Strengths of this study include the large nationally representative sample and the use of professionally obtained height and weight measurements for classifying obesity. This may have avoided inaccuracies associated with self‐reported BMI such as underestimation due to social stigma and social desirability which have affected other studies using self‐reporting.^17^, ^18^ The similar results obtained in sensitivity analyses using waist–hip ratio to classify obesity and removing the overweight category to compare obese individuals with normal/underweight individuals further strengthen our findings. The use of self‐reported ILI as an outcome measure may have captured the community prevalence more accurately than clinical surveillance studies based on those who seek medical care,^21^, ^22^ and it also avoided issues with timing and accuracy of laboratory tests. Nonetheless, using HSE data on ILI had some limitations. HSE questions only asked about one episode of illness so multiple episodes would have been missed. Recall bias, particularly in interviews taking place many months after illness due to the rolling nature of the survey, may have affected ILI reporting.^16^ Mild symptoms, which were a typical feature of many infections in the 2009 influenza pandemic, may not have been attributed to ILI by participants. There is also a risk that media coverage of the pandemic may have resulted in a change in reporting behaviour among participants. Although validating our measure of ILI against other definitions such as the European Centre for Disease Prevention and Control definition would have enhanced results, it was not possible with the limited ILI data collected in the HSE. The HSE does, however, provide a unique breadth of insight into population‐level general health and social issues, which exceeds that of most other sources, and allows consideration of a range of potential confounding factors.

The use of weighting in analyses helped to ensure that participants selected were representative of the population at both regional and national level. There may, however, have been residual selection biases: institutionalized populations, those with mental disabilities, children without parental consent and those who could not speak English were excluded from participation. As institutionalized populations are more likely to be older and are potentially less healthy than those living as private residents in England, they may be more likely to experience ILI. Exclusion of those with mental disabilities or children less than 16 years without parental consent may also have underestimated the frequency of ILIs and may limit generalizability of results to these groups. It was not possible to examine the interaction between pregnancy and obesity as risk factors for ILI. Pregnant women were excluded due to difficulties with interpreting BMI in this group, but they only made up 0.65% of the HSE 2010 core population from which our sample was drawn.

Overall in 2010, data from our study showed that around a quarter (24.7%) of the English population was obese (BMI >30 kg/m^2^). Among those who were obese, 12.8% experienced ILI between May and December 2009. Although this study found no evidence that obesity was associated with an increase in likelihood of self‐reporting ILI symptoms during the 2009 H1N1 pandemic in England, there is some suggestion from other studies that a higher proportion of obese people who become infected with influenza progress to severe disease than those with a normal weight.^1^, ^2^ While the mechanisms of association between obesity and severe influenza are not well understood, murine models provide some evidence that obesity may delay innate immune activation in response to influenza infection and therefore lead to a suboptimal adaptive immune response.^23^ Obese people may therefore be a sensible target group for antiviral drugs when they do develop ILI. There are also unanswered questions about the use of influenza vaccine in obesity. In October 2014, the Joint Committee on Vaccination and Immunisation in the United Kingdom recommended influenza vaccination to reduce the chances of complications following influenza infections for those who are morbidly obese (BMI >40 kg/m^2^).^24^ This has not yet been adopted into the UK vaccine schedule, so currently, people with BMI >40 kg/m^2^ are only vaccinated if they meet other vaccine criteria such as diabetes.^24^, ^25^ Further studies of the role of obesity in a larger population, preferably using active prospective surveillance of ILI combined with laboratory reporting would help to minimize recall bias and improve outcome measurements. This research will be invaluable for informing healthcare planning, guiding targeting of resources and informing governments to ensure a proportionate response to future influenza pandemics.

## Competing Interests

All authors reported that they have no competing interest to declare.

## Financial Statement

This work had no specific funding.
