# Supplementary figures and images for: Investigating obesity as a risk factor for influenza‐like illness during the 2009 H1N1 influenza pandemic using the Health Survey for England
Source: Influenza Other Respir Viruses. 2016 Aug 20;11(1):66–73. doi: 10.1111/irv.12420 (PMC5155645; doi:10.1111/irv.12420)

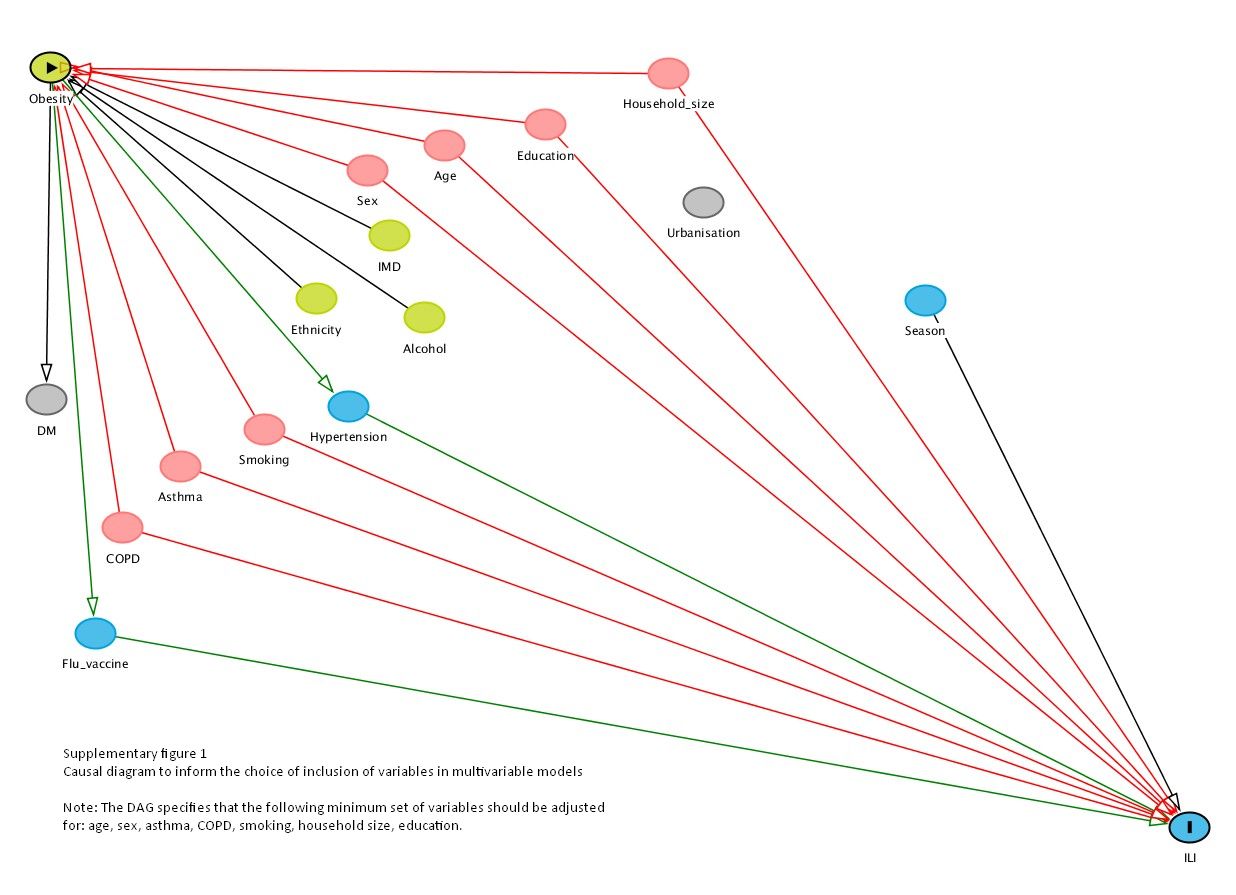

Supplement: Supplementary file 1 [file IRV-11-66-s001.jpg]
